# Thermo-Optical Control of Raman Solitons in a Functionalized Silica Microsphere

**DOI:** 10.3390/mi13101616

**Published:** 2022-09-27

**Authors:** Elena A. Anashkina, Maria P. Marisova, Alexey V. Andrianov

**Affiliations:** 1 Institute of Applied Physics of the Russian Academy of Sciences, 46 Ul’yanov Street, 603950 Nizhny Novgorod, Russia; 2 Advanced School of General and Applied Physics, Lobachevsky State University of Nizhny Novgorod, 23 Gagarin Ave., 603950 Nizhny Novgorod, Russia

**Keywords:** silica microsphere, optical microcavity, whispering gallery modes, optical frequency comb, Raman soliton, thermo-optical control

## Abstract

The investigation of optical microcavity solitons is in demand both for applications and basic science. Despite the tremendous progress in the study of microresonator solitons, there is still no complete understanding of all features of their nonlinear dynamics in various regimes. Controlling soliton properties is also of great interest. We proposed and investigated experimentally and theoretically a simple and easily reproducible way to generate Raman solitons with controllable spectral width in an anomalous dispersion region in a functionalized silica microsphere with whispering gallery modes (WGMs) driven in a normal dispersion regime. To functionalize the microsphere, coating (TiO_2_ + graphite powder) was applied at the pole. The coating is used for effective thermalization of the radiation of an auxiliary laser diode launched through the fiber stem holding the microsphere to control detuning of the pump frequency from exact resonance due to the thermo-optical shift of the WGM frequencies. We demonstrated that the thermo-optical control by changing the power of an auxiliary diode makes it possible to switch on/off the generation of Raman solitons and control their spectral width, as well as to switch Raman generation to multimode or single-mode. We also performed a detailed theoretical analysis based on the Raman-modified Lugiato–Lefever equation and explained peculiarities of intracavity nonlinear dynamics of Raman solitons. All experimental and numerically simulated results are in excellent agreement.

## 1. Introduction

Soliton optical frequency combs generated in microcavities with whispering gallery modes (WGMs) have been attracting great attention in recent years [1,2,3,4,5,6,7]. They have a huge number of applications, e.g., in spectroscopy, LIDAR, massively parallel coherent telecommunications, optical frequency synthesizers, low-noise microwave generation, and so on [1]. The first demonstration of bright dissipative Kerr solitons in microcavities was reported in [2]. After that, diverse soliton regimes were investigated, e.g., dark solitons [3], platicons [4], bright-dark soliton pairs [5], Raman solitons [7], and many others. Cavity Raman (Stokes) solitons are generated at the central frequency shifted from the pump frequency by the value close to the frequency of maximum Raman gain. Raman combs are characterized by low noise [8,9] and have no constant temporal background, contrary to dissipative solitons near the pump, which may be convenient for applications. 

It should be noted that the formation of Raman solitons at the Stokes frequency was first theoretically predicted and investigated in optical fibers by V.A. Vysloukh and V.N. Serkin [10,11], and then experimentally implemented by E.M. Dianov with co-authors [12]. After that, the influence of the Raman nonlinearity on the nonlinear soliton dynamics in optical fibers was actively studied in many works (see, for example, book [13] and references therein and pioneering works [14,15,16,17,18]). At present, Raman solitons in optical fibers have been studied very well [13]. Raman solitons in microcavities have some distinctive features from the Raman solitons in fibers. For microcavity solitons, such features are the discrete spectrum of the resonator modes, resonant continuous-wave pumping, strong influence of the detuning of the pump frequency from the resonance, and intricate interaction between the pump-driven Kerr comb formed near the pump frequency and the long-wavelength Raman soliton. To date, there are only a few works exploring microcavity Raman solitons in different regimes. A Raman soliton held in the potential of a dissipative Kerr soliton belonging to another mode family was discovered in a microcavity fabricated from silica on silicon [7]. Both solitons (main and Raman ones) were located in the anomalous dispersion region [7]. Raman solitons in two mode families in the anomalous dispersion region driven by a pump in the normal dispersion region were experimentally observed in a silica microsphere [19]. A Raman soliton in a silica microrod driven by a CW pump was studied in [20]. However, the nonlinear dynamics and the possibility of controlling the parameters of Raman solitons have not yet been fully explored, especially with allowance for thermo-optical effects leading to WGM frequency shifts.

It is well known that thermo-optical WGM frequency shifts occur in microcavities due to partial thermalization of pump power [21]. This effect has to be taken into account and special means are needed to initiate nonlinear optical (or laser) conversion of narrow-band pump in microcavities, especially for fixed-wavelength pump lasers. Therefore, various passive and active methods are used to start the generation of optical frequency combs or other nonlinear (or laser) processes [22]. However, the thermo-optical effect, if properly controlled by auxiliary devices, can also be used to start and control pump conversion. For example, the thermo-optical control of soliton frequency comb generation in a silicon nitride microring resonator using a microheater was reported in [23]. Recently, we proposed to use a violet-blue auxiliary laser diode to start lasing in an Er:tellurite microcavity [24]. A tellurite glass microsphere absorbed diode light at a wavelength of 405 nm, resulting in its efficient thermalization [24]. In our work, for Raman soliton generation, we use a silica microsphere with a relatively low absorption coefficient in visible and near-IR ranges. For effective thermo-optical control of WGM frequency shifts using an auxiliary laser diode, we propose to functionalize a microsphere by applying a coating near its pole. We propose to use cheap non-toxic components, such as titanium dioxide (TiO_2_) powder in silicone grease mixed with graphite powder, widespread in daily life. Graphite efficiently absorbs and thermalizes near-IR light at 0.98 µm. TiO_2_ in silicone grease is a basis with a high thermal conductivity desirable for uniform diode power thermalization. Here, we investigate both experimentally and theoretically the features of Raman soliton generation with controllable spectral width in the anomalous dispersion region driven by a CW pump in the normal dispersion regime in a functionalized silica microsphere. Particularly, we study the influence of detuning of the pump frequency from the exact resonance on the parameters of Raman solitons. We demonstrate thermo-optical start and control of Raman soliton generation for the first time, to the best of our knowledge.

## 2. Materials and Methods

### 2.1. Fabrication of Functionalized Microspheres

First, we made a microsphere with a diameter of about 180 µm on a fiber stem from a commercial silica fiber using optical fiber splicer hardware with specially developed software [25,26]. The technique is based on multiple melting of a tapered fiber end using an electric arc discharge to form a sphere [25,26]. This technique allows us to fabricate microcavities with reproducible parameters [26] and controllable dispersion via controlling the sphere diameter [27]. This microcavity with free spectral range FSR = 354 GHz supports the propagation of WGMs near the equator. The microcavity size was purposely chosen to provide normal dispersion at 1.46 µm corresponding to the pump wavelength and anomalous dispersion for the wavelengths >1.53 µm where Raman solitons were generated. The target microcavity diameter was found by numerical modeling. (for details please see [27]). The larger the microsphere, the shorter the zero-dispersion wavelength (ZDW) is. For example, for diameters of 100, 180, 300, and 400 µm, the calculated ZDWs are 1.69, 1.53, 1.45, and 1.41 µm, respectively. For wavelengths longer than the ZDW, the dispersion is anomalous.

**Figure 1:**
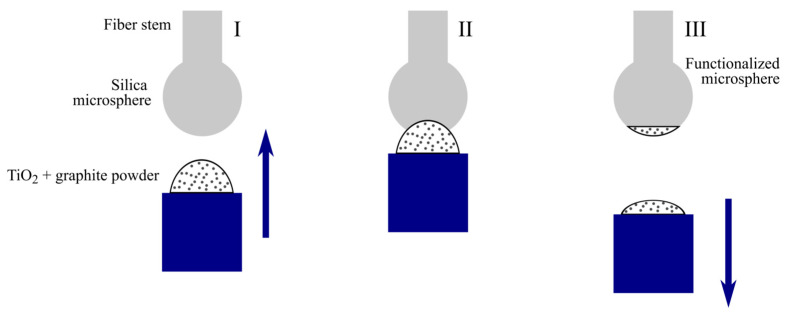
Process of microsphere coating with TiO_2_ mixed with graphite powder.

Next, the produced microsphere was functionalized by a coating made of titanium dioxide TiO_2_ powder in silicone grease mixed with graphite powder. Graphite powder efficiently absorbs and thermalizes near infrared light; TiO_2_ in a silicone grease is a basis with a high thermal conductivity. This coating was precisely applied near the microsphere pole opposite to the fiber stem. The controlling light at 0.98 µm was coupled into the microsphere through the fiber stem and then its thermalization occurred near the pole in the coating. The purpose of the coating was effective thermalization of the radiation of the auxiliary laser diode for thermo-optical control of pump detuning from the exact resonance due to thermo-optical shifts of WGM frequencies. The process of microsphere coating is depicted in Figure 1. The diameter of the coated area was about 40 µm. This coating is located sufficiently far from the WGM region and does not affect Q-factor, FSR, or dispersion. 

### 2.2. Measuring Q-Factors

**Figure 2:**
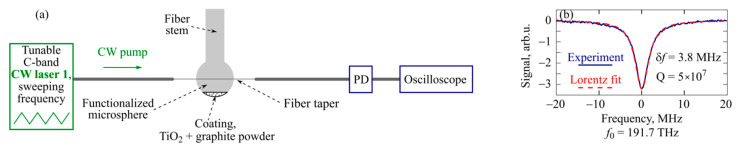
(**a**) Experimental setup for measuring Q-factors of microcavities. (**b**) Typical resonance curve of produced functionalized microsphere.

The Q-factors of the produced functionalized microspheres were measured according to the scheme in Figure 2a. We used a narrow-band pump laser (CW laser 1) with a linewidth of 10 kHz, whose frequency was swept at a rate of 15 GHz/s with an amplitude of 60 GHz around the center frequency *f*_0_ = 191.7 THz (~1.56 µm). The laser radiation was launched into the functionalized microspheres using a silica fiber taper with a waist diameter of about 2 µm. The resonances were measured using an oscilloscope. A typical resonance curve with a width of 3.8 MHz corresponding to a loaded Q-factor of 5 × 10^7^ is shown in Figure 2b. Note that microspheres without coating have very similar Q-factors. So, the coating indeed does not affect the Q-factor, since it is far from the equator near which WGMs propagate. 

### 2.3. Numerical Simulations

For the calculation of FSR, WGM fields, WGM effective volumes, and microcavity dispersion, we used the well-developed approach based on numerical solution of the characteristic equation [28]. 

Steady-state temperature fields were found numerically by the finite element method using COMSOL Multiphysics software. The obtained data were imported into specially developed software simulating frequency shifts of WGMs due to refractive index changes (*dn*/*dT*) and thermal expansion [29]. The temperature was calculated using an axially symmetric model, taking into account the geometric parameters of the functionalized microsphere. We assumed that control light at 0.98 µm was coupled into the microsphere through the fiber stem and then its thermalization occurred near the pole in the coating (TiO_2_ + graphite powder). To model the coating contribution to the process of thermo-optical control, we set the constant temperature *T*_room_ + Δ*T*_coating_ as a boundary condition near the microsphere pole for the area with a diameter of 40 µm, where *T*_room_ is the room temperature and Δ*T*_coating_ is the temperature increase in the coating. On the rest of the surface of the functionalized microsphere and the fiber stem, the boundary condition of natural convection in air was set (standard condition in COMSOL). We also verified that modeling the coating as a heat source at the boundary with a uniformly dissipated power gives results similar to those when an appropriate constant temperature is set. WGM frequency shifts occur for two reasons: (1) refractive index changes in the region where WGM propagates, which depend on the temperature increase averaged over the WGM volume in our model, and (2) changes in microsphere diameter caused by thermal expansion, which depend on the temperature increase averaged over the whole microsphere. We also considered partial thermalization of pump power in the region corresponding to the effective WGM volume near the microsphere equator, which also affects the temperature distribution and WGM shifts. We previously developed the model describing WGM frequency shifts due to pump thermalization and experimentally verified its performance [29]. Thermo-optical control of the WGM frequency shifts achieved due to microsphere functionalizing allows us to control pump frequency detuning from exact resonance in the experiment, which greatly affects the intracavity nonlinear dynamics of radiation and the regimes of pump conversion.

To model nonlinear optical processes in microcavities, the generalized Lugiato–Lefever equation is often used (in contrast to nonlinear optical processes in fibers often described by the generalized nonlinear Schrödinger equation). The basic form of the Lugiato–Lefever equation was first presented in [30]. Here, to simulate the nonlinear dynamics of intracavity radiation leading to Raman soliton generation, we used the Raman-modified Lugiato–Lefever equation in dimensionless form [31,32]:

(1) $$\frac{\partial E\left(t,\tau \right)}{\partial t}=\left[-1-i\Delta +\sum_{k=2}^{4}\frac{B_{k}}{k!}{\left(i\frac{\partial }{\partial \tau }\right)}^{k}\right]E+\sqrt{P}+i\left[\left(1-f_{R}\right)|E{|}^{2}+f_{R}[\Gamma \left(\tau ,\tau_{S}\right)⊗|E{|}^{2}]\right]E,$$

where *E*(*t*,*τ*) is the intracavity field envelope depending on slow time *t* and fast time *τ*; *P* is the pump power; *Γ* is the model Raman response function; *f_R_* is the fraction of the Raman contribution; and ⊗ denotes convolution. The terms in the right-hand side of Equation (1) describe losses, detuning, dispersion, pump, and Kerr and Raman nonlinearities. Normalization was performed as in [32]. 

## 3. Results

### 3.1. Thermo-Optical Control of WGM Frequency Shifts (Simulations)

**Figure 3:**
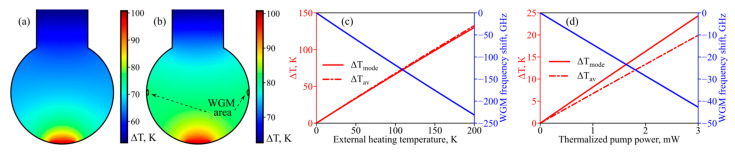
*Numerical simulation.* (**a**) Temperature distribution in functionalized silica microsphere with 180 µm diameter for coating temperature increase Δ*T*_coating_ = 100 K for thermalized pump power 0 mW (**a**) and 2 mW (**b**). (**c**,**d**) Temperature increases averaged over microsphere (red solid line, left axis) and WGM volume (red dash-dotted line, left axis) as well as the corresponding WGM eigenfrequency shift (blue line, right axis) as functions of Δ*T*_coating_ (**c**) and thermalized pump power (assuming Δ*T*_coating_ = 0 K) (**d**).

First of all, we theoretically investigated the possibilities of thermo-optical control of WGM frequencies using coating and an auxiliary laser diode. An example of the simulated temperature field at Δ*T*_coating_ = 100 K is given in Figure 3a. Figure 3b shows an example of the simulated temperature field when, in addition to thermalization of diode radiation in the coating, pump radiation with power *P*_mode_ = 2 mW in the WGM area is partially thermalized. We calculated the temperature increase averaged over the whole microsphere (Δ*T*_av_), temperature increase averaged over the WGM area (Δ*T*_mode_), and the corresponding WGM frequency shifts as functions of external heating Δ*T*_coating_ (Figure 3c). We also checked that for simultaneous heating at the pole by a laser diode and heating in the mode area by a pump laser, the temperature increase is almost additive Δ*T*(*P*_mode_, Δ*T*_coating_) ≈ Δ*T*(*P*_mode_ = 0, Δ*T*_coating_) + Δ*T*(*P*_mode_, Δ*T*_coating_ = 0); the maximum discrepancy is ~2%. The calculated averaged temperature increases Δ*T*_mode_ and Δ*T*_av_ as well as WGM frequency shifts as functions of *P*_mode_ for Δ*T*_coating_ = 0 are plotted in Figure 3d. So, the thermo-optical shifts of WGMs can be comparable with FSR for reasonable experimental parameters and provide great opportunities for controlling detuning. As expected, the influence of heating in the coating is higher than in the mode.

### 3.2. Raman Soliton Generation under Thermo-Optical Control (Experiments)

**Figure 4:**
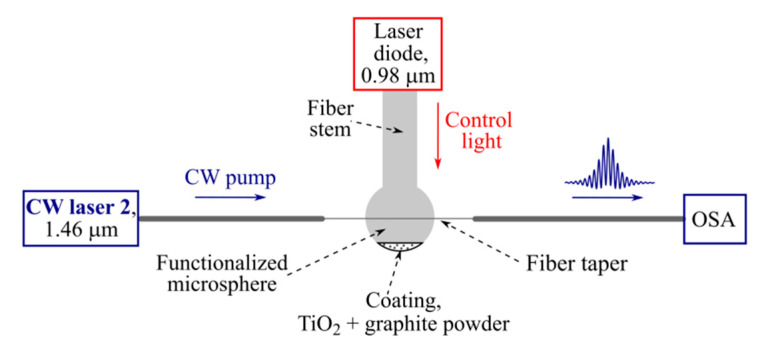
Schematic diagram of experimental setup for Raman soliton generation in functionalized microsphere pumped at 1.46 µm under thermo-optical control by laser diode at 0.98 µm.

We performed a series of experiments according to the scheme in Figure 4. To generate optical frequency combs, we used a narrow-band pump laser with a linewidth < 200 kHz (CW laser 2) at a fixed wavelength of 1.46 µm. To effectively control the detuning of the pump frequency from exact resonance through the WGM thermo-optical shift, we used a laser diode at a wavelength of 0.98 µm. The radiation of the laser diode was fed into the functionalized microsphere through a fiber stem and then thermalized in the coating (TiO_2_ + graphite powder), resulting in microsphere heating. 

**Figure 5:**
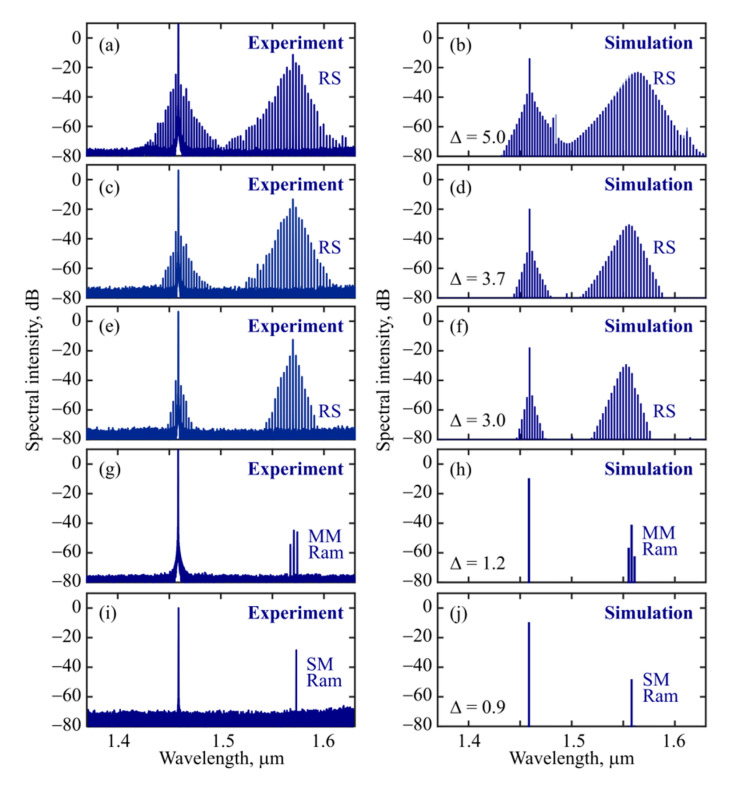
Experimentally measured (left column) and numerically simulated (right column) spectra demonstrating Raman soliton (RS) generation (**a**–**f**), multimode Raman (MM Ram) generation (**g**,**h**), and single-mode Raman (SM Ram) generation (**i**,**j**). Compare pairs (**a**–**j**). Pump power at 1.46 µm is constant 12 mW (before the taper) and power of 0.98 µm auxiliary laser diode is attenuated from 200 mW (at the beginning of the experiment, when there is no Raman generation) to: 80 mW (**a**), 65 mW (**c**), 50 mW (**e**), 20 mW (**g**), and 0 mW (**i**).

At the beginning of the experiments, both the laser diode with maximum power (200 mW in the case under consideration) and CW laser 2 at a fixed wavelength of 1.46 µm were switched on. The WGM frequencies were shifted to longer wavelengths relative to cold resonances due to significant thermalization of diode radiation in the coating and slight thermalization of the pump power in the WGM region. In this case, there was neither generation of optical frequency combs nor Raman generation, since the fixed pump frequency was far from hot resonance. Further, we started reducing the power of the auxiliary laser diode (down to 0), thereby the WGM frequencies were blue-shifted relative to “the hottest” position. The frequency shift was quite significant compared to the FSR in conformity with the calculations presented in Figure 3c. The resonant WGM frequency approached the pump frequency from the long-wavelength side, thus initiating mode pulling by the pump, and the generation of new harmonics due to Raman and Kerr nonlinearities. By changing the power of the laser diode, we effectively controlled the detuning and, thus, the parameters of optical frequency combs. Controlling the diode power allowed us to achieve the regimes of Raman soliton generation in the anomalous dispersion region as well as multimode and single-mode Raman generation (Figure 5, left column). In the case of Raman soliton generation, a weak comb was also formed near the pump in the normal dispersion region. The Raman soliton and the weak comb near the pump belonged to the same mode family. Depending on detuning, we could control the spectral width of the solitons (Figure 5, left column). The corresponding numerical simulation results (Figure 5, right column) are in excellent agreement with the experimental measurements.

**Figure 6:**
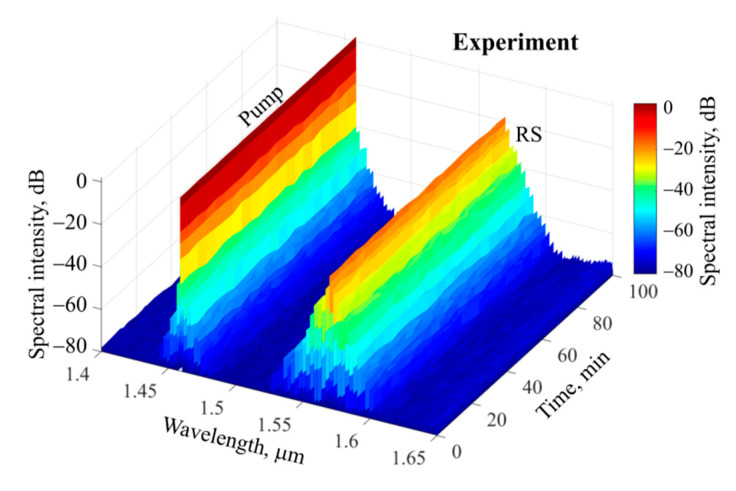
Stable spectra of optical frequency comb measured for 100 min. Experimental parameters are set as for the comb in Figure 5c. RS is Raman soliton.

We tested the stability of the Raman solitons for 100 min. The spectra were recorded every 5 min, and the results are shown in Figure 6. The experimental parameters were set as in Figure 5c. The optical frequency comb possesses an excellent stability, which is very attractive for applications. 

### 3.3. Theoretical Analysis of Raman Soliton Generation

**Figure 7:**
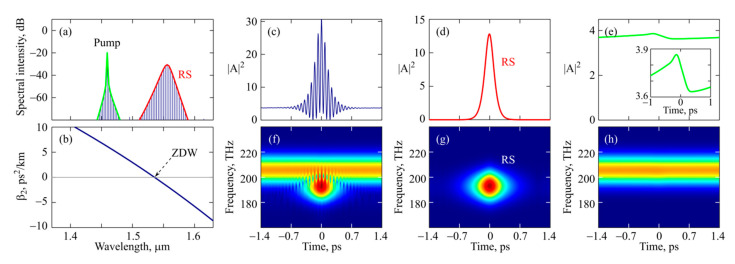
*Numerical simulation*. (**a**) Spectral intensity of optical frequency comb for Δ = 3.7 and *P* = 30. The red line shows spectral envelope for Raman soliton (RS) and the green line shows spectral envelope for weak comb near pump wavelength. (**b**) Dispersion of used microsphere; ZDW is zero dispersion wavelength. Temporal intensities of the entire frequency comb (**c**), RS (**d**), and weak comb near pump wavelength (**e**). Spectrograms of entire optical frequency comb (**f**), RS (**g**) and weak comb near pump wavelength (**h**). Window function for all spectrograms is exp[−*t*^2^/(40 fs)^2^].

Next, we performed a more detailed theoretical analysis of the generated Raman solitons. We considered a typical example of an optical frequency comb with the spectrum shown in Figure 7a (simulated with *P* = 30 and Δ = 3.7). The calculated microsphere dispersion is plotted in Figure 7b. The microsphere was pumped in the normal dispersion region, and the Raman soliton was generated in the anomalous dispersion region. The spectral intensity envelope of the Raman soliton is shown in Figure 7a by the red curve, and the spectral intensity envelope of the weak comb near the pump is shown in the same figure by the green curve. The temporal intensity profile of the total signal is plotted in Figure 7c. The temporal structures of the Raman soliton and the signal near the pump are demonstrated in Figure 7d,e, respectively. The spectrograms for the total signal, the Raman soliton, and near-pump radiation are presented in Figure 7f–h, respectively. To construct them, we used a window function in the form *W =* exp[−*τ*^2^/(40 fs)^2^]. The spectrogram in Figure 7f demonstrates that the soliton has an ideal shape and a flat phase, like the ideal soliton in the classical nonlinear Schrödinger equation [33]. The temporal intensity profile of the signal near the pump does not depend on slow time. Note that the Raman soliton and the pump wave shown in Figure 7c are not phase-locked. The fringes “roll” during the evolution. The Raman soliton also affects the near-pump signal and helps to hold the temporal structure in Figure 7e through cross-phase modulation. We confirmed this fact in modeling by artificially adding high loss at wavelengths longer than 1.52 µm, which led to Raman soliton suppression and destruction of the near-pump weak comb. 

**Figure 8:**
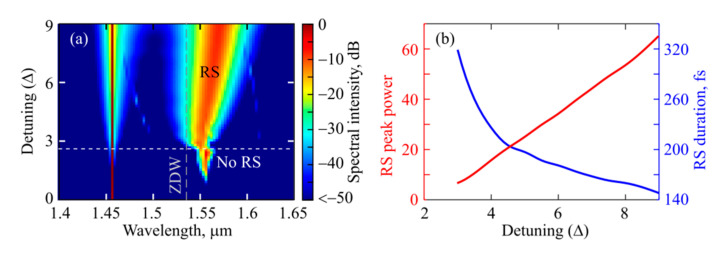
*Numerical simulation*. (**a**) Spectral intensities as a function of detuning for *P* = 30. Horizontal dashed line shows the threshold for Raman soliton (RS) formation. (**b**) RS peak power (red line, left axis) and RS FWHM duration (blue line, right axis) as functions of detuning.

Finally, we numerically considered different values of detuning and simulated the comb spectra as a function of Δ at a constant dimensionless pump power *P* = 30 (Figure 8a). It was found that stable Raman solitons can form only within a certain detuning range of about 2.7 < Δ < 9. At low detuning values (0.7 < Δ < 2.7), mode locking does not occur, but single-mode or multi-mode Raman generation can be observed, which agrees with the experimental data (see Figure 5g–j). When Δ < 0.7, only CW intracavity radiation at the pump wavelength is observed. The explanation is very simple. It is well known that in the CW regime the intracavity power depends on Δ, while with a gradual increase in Δ, the intracavity power increases (until Δ < *P*) [31,34]. At a certain value of Δ ≈ 0.7, the threshold of Raman generation is reached. When the threshold is slightly exceeded, the Raman generation is single-mode (compare Figure 5g,h). As Δ is increasing with a noticeable excess of the intracavity power relative to the threshold, multimode Raman generation develops, but the total power in different harmonics is insufficient to form a soliton (the harmonics are not mode-locked). With a still higher increase in Δ > 2.7, the intracavity power at the pump frequency reaches values at which the generated power in the Raman harmonics is sufficient to form a soliton. Further, as the detuning increases, the peak power *P*_RS_ of the Raman soliton increases, the spectrum broadens, and its FWHM (full width at half maximum) duration *T*_RS_ decreases (Figure 8b). In this case, the central wavelength of the soliton λ_RS_ slightly increases (Figure 8a), which is explained by the Raman amplification of low-frequency harmonics by higher-frequency harmonics. Note that a similar effect is also known for dissipative Kerr solitons formed near the pump frequency, when the central soliton frequency shifts to longer wavelengths relative to the pump with increasing detuning due to non-zero Raman gain at low frequencies [32,35]. We verified that our solitons satisfy the condition γ⸱*P*_RS_·(*T*_RS_/1.763)^2^/|*B*_2_(λ_RS_)| ≈ 1 like for classical solitons in the nonlinear Schrödinger equation [33]. In our system, the round-trip losses are completely compensated by the Raman gain for the Raman soliton. When the detuning Δ ≈ 9 is reached, the Raman soliton begins to exhibit breather behavior, which requires a separate study and is beyond the scope of this work.

## 4. Discussion

We proposed and investigated both experimentally and theoretically the generation of stable Raman solitons at a wavelength of 1.57 µm in the anomalous dispersion region in a functionalized silica microsphere with WGMs driven in the normal dispersion regime at a fixed pump wavelength of 1.46 µm. The microsphere was functionalized by applying coating (TiO_2_ + graphite powder) at the pole far from WGMs. The coating effectively absorbed and thermalized the radiation of an auxiliary laser diode at a wavelength of 0.98 μm fed through the fiber stem to effectively control the detuning of the pump frequency from exact resonance due to the thermo-optical shift of the WGM frequencies. The coating did not affect the Q-factor (5 × 10^7^) and dispersion. We demonstrated that due to thermo-optical control by changing the power of the auxiliary diode, it is possible to switch on/off the generation of Raman solitons and control their spectral width, as well as switch Raman generation to multimode or single-mode.

Note that the proposed system is relatively simple, cheap, and easily reproducible. In the proposed concept, we only need a narrow-band laser at a fixed wavelength and a standard auxiliary laser diode. The coating is based on cheap, widely used non-toxic components. The microsphere is made of a standard silica fiber using a fiber splicer. The dispersion is controlled by changing the microsphere diameter, which makes it possible to adjust the parameters to obtain a Raman soliton at a certain wavelength. The developed approach can be used to generate Raman solitons at different central wavelengths, but a pump wavelength should be in the normal dispersion region and the Raman soliton red-shifted by ~13 THz should be in the anomalous dispersion region. Moreover, the proposed method of microsphere functionalization for thermo-optical control of WGM frequency shifts, and hence detuning, can be used to switch on/off other regimes of the optical frequency comb generation and to easily control parameters of converted light. 

All our experimental and numerically simulated results are in excellent agreement. We also performed a detailed theoretical analysis that helps to better understand the ongoing processes. Our numerical simulations based on the Raman-modified Lugiato–Lefever equation revealed that Raman solitons can exist only in a certain range of detunings (at a specific pump power). In this case, the Raman gain completely compensates for the losses, and the shape and properties of the solitons are the same as for classical solitons in the nonlinear Schrödinger equation. Moreover, when Raman solitons are generated, a weak optical frequency comb is generated near the pump, which interacts with the soliton via phase cross-modulation. The temporal structure of this comb does not depend on slow time, but the soliton and the pump wave are not phase-locked. Unlike dissipative Kerr solitons generated in microcavities driven in the anomalous dispersion regime, Raman solitons have no constant background. In addition, the initiation of dissipative Kerr solitons is not an easy task, while Raman solitons can be started easier, which may be important for applications.

## Data Availability

Data underlying the results presented in this article may be obtained from the authors upon reasonable request.
